# Postnatal growth in small vulnerable newborns: a longitudinal study of 2 million Brazilians using routine register-based linked data

**DOI:** 10.1016/j.ajcnut.2023.12.009

**Published:** 2023-12-20

**Authors:** Aline S. Rocha, Rita de Cássia Ribeiro-Silva, Juliana F.M. Silva, Elizabete J. Pinto, Natanael J. Silva, Enny S. Paixao, Rosemeire L. Fiaccone, Gilberto Kac, Laura C. Rodrigues, Craig Anderson, Mauricio L. Barreto

**Affiliations:** 1 Center of Data and Knowledge Integration for Health (CIDACS), Oswaldo Cruz Foundation, Salvador, Brazil; 2 School of Nutrition, Federal University of Bahia (UFBA), Salvador, Brazil; 3 Institute of Collective Health, Federal University of Bahia (ISC/UFBA), Salvador, Brazil; 4 Health Sciences Center, Federal University of Recôncavo da Bahia, Santo Antônio de Jesus, Brazil; 5 ISGlobal, Hospital Clínic. Universitat de Barcelona, Barcelona, Spain; 6 Department of Statistics, Federal University of Bahia (UFBA), Salvador, Brazil; 7 Nutritional Epidemiology Observatory, Josué de Castro Nutrition Institute, Federal University of Rio de Janeiro, Rio de Janeiro, Brazil; 8 Faculty of Epidemiology and Population Health, London School of Hygiene and Tropical Medicine, London, United Kingdom; 9 School of Mathematics and Statistics, University of Glasgow, Scotland, United Kingdom

**Keywords:** growth, low–birth weight newborn, preterm birth, small-for-gestational age

## Abstract

**Background:**

Preterm, low–birth weight (LBW) and small-for-gestational age (SGA) newborns have a higher frequency of adverse health outcomes, including linear and ponderal growth impairment.

**Objective:**

To describe the growth trajectories and to estimate catch-up growth during the first 5 y of life of small newborns according to 3 vulnerability phenotypes (preterm, LBW, SGA).

**Methods:**

Longitudinal study using linked data from the 100 Million Brazilian Cohort baseline, the Brazilian National Live Birth System (SINASC), and the Food and Nutrition Surveillance System (SISVAN) from 2011 to 2017. We estimated the length/height-for-age (L/HAZ) and weight-for-age *z*-score (WAZ) trajectories from children of 6–59 mo using the linear mixed model for each vulnerable newborn phenotype. Growth velocity for both L/HAZ and WAZ was calculated considering the change (Δ) in the mean *z*-score between 2 time points. Catch-up growth was defined as a change in *z*-score > 0.67 at any time during follow-up.

**Results:**

We analyzed 2,021,998 live born children and 8,726,599 observations. The prevalence of at least one of the vulnerable phenotypes was 16.7% and 0.6% were simultaneously preterm, LBW, and SGA. For those born at term, all phenotypes had a period of growth recovery from 12 mo. For preterm infants, the onset of L/HAZ growth recovery started later at 24 mo and the growth trajectories appear to be lower than those born at term, a condition aggravated among children with the 3 phenotypes. Preterm and female infants seem to experience slower growth recovery than those born at term and males. The catch-up growth occurs at 24–59 mo for males preterm: preterm + AGA + NBW (Δ = 0.80), preterm + AGA + LBW (Δ = 0.88), and preterm + SGA + LBW (Δ = 1.08); and among females: term + SGA + NBW (Δ = 0.69), term + AGA + LBW (Δ = 0.72), term + SGA + LBW (Δ = 0.77), preterm + AGA + LBW (Δ = 0.68), and preterm + SGA + LBW (Δ = 0.83).

**Conclusions:**

Children born preterm seem to reach L/HAZ and WAZ growth trajectories lower than those attained by children born at term, a condition aggravated among the most vulnerable.

## Introduction

It is well established that infants who were born preterm (born before 37 wk of gestation) [1], small-for-gestational age (SGA, newborns weighing below the 10th percentile for gestational age) [2] (a proxy for intrauterine growth restriction), and of low–birth weight (LBW, birth weight below 2500 g) [3] have a higher risk of several adverse health outcomes during childhood such as neonatal infections, developmental delays, chronic health disorders, and growth impairment [[4], [5], [6], [7], [8]].

Although described as distinct conditions, preterm birth and SGA can coexist and carry a particularly high risk of serious clinical complications, requiring intensive neonatal care or leading to death compared with infants with either characteristic alone [9]. Ashorn et al. [10], in the *Lancet Vulnerable Newborn* series, highlighted the importance of defining vulnerable newborn phenotypes, combining preterm, SGA, and LBW, to provide a better scientific basis for the development of national and global commitments to provide a healthy start in life for all the newborns [10].

Preterm birth, SGA, and LBW rates are high worldwide [[11], [12], [13]]. Also, it was estimated that out of 135 million live births in 2020, 35.3 million (26.2%) were small vulnerable newborns (SVNs), defined as any infant born preterm, or SGA, or both preterm and SGA [13]. In Brazil, a population-based study found that the prevalence of preterm birth was 9.4%, SGA was 9.2%, and LBW was 9.6%. However, 18% of newborns were classified as SVNs, combining 3 phenotypes (preterm, SGA, and LBW). The SVNs presented a risk of mortality 62 times greater than infants born at term who were neither LBW or SGA [14]. Size at birth is an important indicator of fetal, neonatal, child, and adult health [15].

The longitudinal growth monitoring has proved to be a valuable and cost-effective tool in primary health care [16], because it can identify deviations that could compromise children’s health at an early stage. However, there is still a lack of studies that assess growth trajectories on SVNs, particularly in low- and middle-income countries such as Brazil.

Thus, we aimed to describe the growth trajectories and estimate catch-up growth during the first 5 y of life of SNVs, according to 3 vulnerability phenotypes (preterm, LBW, SGA), using data from the Center for Data and Knowledge Integration for Health (CIDACS) Birth Cohort. The findings are expected to identify sensitive time periods for interventions to support this vulnerable population, contributing to the country’s achievement of the 2025 Global Nutrition Targets related to the reduction of child stunting (target 1) and low–birth weight (target 2), and the Sustainable Development Goals (SDGs) for 2030 focused on ensuring a healthy life and promoting well-being for everyone of all age groups (SDG 1) and the eradication of hunger and all forms of malnutrition (SDG 2) [17,18].

## Methods

### Study design and population

This population-based longitudinal study used data from the CIDACS Birth Cohort, linking data from the 100 Million Brazilian Cohort, the National System of Live Births in Brazil (SINASC), and the Food and Nutrition Surveillance System (SISVAN). Data consist of children aged 6–59 mo, born from January 1, 2011 to December 31, 2015, and followed up until December 31, 2017. This study adhered to the Reporting of studies Conducted using Observational Routinely collected Data (RECORD) statement.

### Data source

The CIDACS Birth Cohort is a dynamic cohort created by the Center for Data and Knowledge Integration for Health (CIDACS) [19]. The study population was composed using data linked from 3 different Brazilian databases [20]. The 100 Million Brazilian Cohort baseline was established using administrative records from >114 million individuals aged 16 y or older, whose families have monthly income ≤ 3 minimum wages (∼750 USD and applied for social assistance through the Unified Register for Social Programmes (Cadastro Único para Programas Sociais, CadUnico). The 100 Million Brazilian Cohort baseline contains demographic and socioeconomic information on all family members, which is provided by a designated representative of the family. A detailed information on the 100 Million Brazilian Cohort is available in another publication [22].The Live Birth Information System (SINASC, Sistema de Informação sobre Nascidos Vivos) is an information system that records data from the Declaration of Live Birth (DLB), a legal document completed by the health worker who attended the delivery. In accordance with the Brazilian Ministry of Health, the DBL must be completed in accordance with the Brazilian Ministry of Health (MS)’s Instruction Manual for Completing the Declaration of Live Birth [23]. Brazil has ∼3 million births a year. A total of 44,485,267 births were recorded in SINASC between 2001 and 2015. An evaluation of the national birth registration systems found that >94% of Brazilian live births are registered in SINASC [24,25]. It includes information on the mother (e.g., mother’s name, place of residence, age, marital status, education), pregnancy (e.g., length of gestation, type of delivery), newborn (e.g., singleton, multiples, birth weight; the presence of congenital anomalies, and gestational age) [23]. SINASC is considered to have adequate quality, acceptable, representative, opportune, and stable data. These data are well suited to fulfill the intended purpose: to support maternal and child care planning [26].

The Food and Nutrition Surveillance System (SISVAN, Sistema de Vigilância Alimentar e Nutricional) was designed to record anthropometric (e.g., weight, height) and dietary intake data at all stages of life of individuals who use primary public health services, including the nutritional status of children whose health is being tracked as part of the Bolsa Família conditional cash transfer program. Health care professionals routinely collect and enter anthropometric data into the system [27]. The technical norms for the collection and analysis of these data in public health services were established by the Brazilian Ministry of Health [28]. SISVAN has shown an increasing trend in the population target coverage, reaching 45.4% in 2017. The completeness of the date of birth, weight, and height corresponded to almost 100% in the period 2008–2017 [29]. During this same period, SISVAN followed per year an average of 3.6 million children younger than 5 y [30]. The main objective of SISVAN is to inform the evaluation and development of public health nutrition policies [31].

### Linkage process

We linked the 3 databases used in this study using 2 approaches: *1)* deterministic linkage – performed between the 100 Million Brazilian Cohort baseline and SISVAN because both data sets contain the Social Identification Number (SIN), a unique identifier assigned to each individual; and *2)* nondeterministic linkage based on the similarity index – the SINASC live birth records and the cohort baseline were linked using the following variables of the mother at delivery: full name, date of birth (or age in completed years when the date of birth was missing), and the municipality of residence. This method also linked the cohort baseline with a subset of individuals in the SISVAN who did not have SIN. The linkage process used CIDACS-record linkage (RL), a novel RL tool developed to link large-scale administrative data sets at CIDACS [32]. Linkage procedures were carried out at CIDACS in a strict data protection environment and followed ethical and legal standards [19].

### Inclusion and exclusion criteria

**FIGURE 1:**
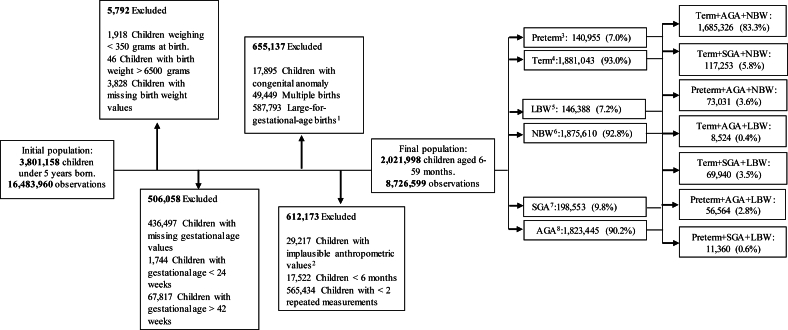
Study population flow diagram, 2011–2017. ^1^Live births large for gestational age (LGA) > 90th percentile by INTERGROWTH-21st); ^2^records with biologically implausible *z*-scores according to the WHO cutoffs for weight-for-age (WAZ) (*z*-scores < −6 and > 5), length/height for age (L/HAZ) (*z*-scores < −6 and > 6); ^3^preterm birth – gestational age < 37 completed weeks; ^4^term-gestational age between 37–42 weeks; ^5^low birth weight (LBW) – birth weight < 2500 g; ^6^normal birth weight (NBW) – birth weight between 2500–6500 g; ^7^small for gestational age (SGA) – birth weight for gestational age <10th percentile of the INTERGROWTH-21st; ^8^adequate for gestational age (AGA) – birth weight for gestational age between the 10th and 90th percentiles.

Children aged 6–59 mo, with 2 or more weight and height measurements evaluated between 2011 and 2017 were included in the study. We excluded all children with missing data on birth weight and gestational age; records with birth weight < 350 and > 6500 g, considering survival [33] and biological implausibility limits; with gestational age at birth < 24 and ≥43 wk, for whom it is not possible to calculate the size for gestational age according to INTERGROWTH-21st [34]; multiple births; live births with a congenital anomaly; and large for gestational age (LGA, newborns weighing above the >90th for gestational age) [2]. We also excluded children with biologically implausible values of weight and height according to the WHO cutoffs for weight-for-age *z*-scores (WAZ < −6 and >5) and length/height-for-age *z*-scores (L/HAZ < −6 and >6) [35]; and with inconsistencies in the height variable, i.e., the negative difference between 2 subsequent ordered measures (1,519,666 negative height observations excluded) (Figure 1).

### Study variables

LBW was defined as birth weight < 2500 g, normal birth weight (NBW) as birth weight between 2500 and 4500 g, preterm birth as gestational age < 37 completed weeks, term as gestational age between 37 and 42 wk, small-for-gestational age (SGA) defined as birth weight for gestational age (in completed weeks) <10th percentile of the INTERGROWTH-21st distribution by gender, and adequate-for-gestational age (AGA) as birth weight for gestational age (in completed weeks) between the 10th and 90th percentiles [34].

Subsequently, children were classified according to 6 mutually exclusive phenotypes based on LBW, preterm birth, and SGA combinations. The phenotypes are *1)* term + AGA + NBW; *2)* term + SGA + NBW; *3)* preterm + AGA + NBW; *4)* term + AGA + LBW; *5)* preterm + AGA + LBW; and *6)* preterm + SGA + LBW. Term + AGA + NBW was used as the reference group. We reclassified the phenotypes into a binary variable: those not SVNs (term + AGA + NBW) and those SVNs (those with at least one of the phenotypes, i.e., preterm birth, SGA, and LBW).

Weight (kg) and length/height (cm) data were obtained from SISVAN records for children younger than 5 y. Standardized measures (L/HAZ and WAZ) were calculated according to the WHO Child Growth Standards [36], using the STATA igrowp package available at https://www.who.int/tools/child-growth-standards/software.

For preterm infants, these measurements were obtained using corrected age (CA), i.e., calculating the difference between GA at birth and gestational duration at term (40 wk), subtracting this difference from postnatal age: CA = postnatal age – [40 − GA at birth (weeks)]/4 [37]. We used corrected age to assess the growth trajectory of preterm infants ≤24 or even 36 mo (for extremely preterm infants [<28 gestational weeks]) of postnatal age [37,38] to obtain the real expectation for each child without underestimating the preterm infant when confronted with children born at term [39].

The following covariates, obtained from the 100 Million Brazilian Cohort baseline and SINASC, were considered in the descriptive analyses: region of residence (North, Northeast, Southeast, South, Midwest), residence area (urban; rural), household overcrowding (no: ≤2 inhabitants per room; yes: >2 inhabitants per room), maternal race (White, Parda/Brown, Black, Indigenous, Asian), maternal education (<3, 4–7, 8–11, ≥12 y of education), marital status (married or in a stable relationship; single, divorced, or widowed); (number of prenatal visits (none; 1–3; 4–7, ≥7 visits), maternal age at delivery (14–19; 20–34; and 35–49 y), number of previous pregnancies (none, 1–3, ≥4 pregnancies), type of delivery (vaginal or cesarean), and gender of the newborn (male or female). Household overcrowding was calculated by dividing the number of individuals living in the house by the number of rooms. The variable related to maternal race (skin color) was derived from the SINASC. In Brazil, maternal race is self-declared and encompasses 5 categories (Black, Parda/Brown, Indigenous, Asian descent, and White) [40].

### Statistical analysis

To calculate the prevalence of preterm birth, LBW, SGA, and SNV, we divided the number of newborns with each phenotype by the total number of live births included in the study, multiplying by 100. Socioeconomic, maternal, and birth characteristics were summarized using frequency distributions. The *χ*^2^ compared the characteristics between the “not small vulnerable” and “small vulnerable” phenotype groups. Mean WAZ and L/HAZ and respective SD values were estimated for each vulnerable newborn phenotype.

We used the Broken-stick model, proposed by Van Buuren [41], for modeling the growth trajectories of L/HAZ and WAZ through a combination of linear segments with different slopes [41]. This model approach has been recommended for assessing irregular individual trajectories and standardized *z*-score data, providing easily interpretable estimates of childhood growth trajectories and good fit for large-scale data sets [42]. In summary, Broken-stick is a linear mixed model approach using second-order linear B-splines, where knots represent change points in the growth trajectory and consider the relative position of each time within a prespecified time interval [43]. Four knots (*K* = 4) located at 6, 12, 24, and 59 mo were selected to model WAZ and L/HAZ based on the inflection points of the WHO Child Growth Standard curves [44] and the convergence of the models. In addition, models were constructed separately by gender to accommodate gender-specific growth patterns and vulnerability phenotypes. The model does not consider loss to follow-up as the child’s age increases. The goodness of fit of the models was evaluated with the visual representation of the observed compared with the predicted values [43]. Graphics account 10% of children in each group, according to gender and vulnerability phenotypes (Supplemental Material).

The growth velocity for WAZ and L/HAZ was calculated by considering the change (Δ) in the mean *z*-score between 2 time points, i.e., 6–12, 12–24, and 24–59 mo. Catch-up growth, defined as a change in *z*-score > 0.67 at any time during follow-up, is characterized by a faster-than-expected growth rate after a period of slow or absent growth [45].

Data processing and descriptive analyses were performed using Stata version 15.1 (Stata Corporation), and growth curves were performed in R version 3.6.0 software (R Foundation for Statistical Computing) [46] using the "brokenstick" package [43].

### Supplementary analysis

Additional analyses were performed on a subdata set to evaluate weight-for-length/height *z*-scores (WLZ/WHZ) trajectories in children younger than 5 y. WLZ/WHZ were also calculated based on the WHO Child Growth Standards [36]. These additional models followed the same method as the main analyses. More information about the analytical sample and approach can be found in Supplemental Material. We also conducted analyses without excluding the negative heights (biologically implausible) to confirm the consistency of our initial findings (Supplemental Material).

### Ethical approval

This research was approved by the research ethics committee at the Institute of Collective Health, Federal University of Bahia (ISC-UFBA) (reference numbers 41695415.0.0000.5030 and 18022319.4.0000.5030) and School of Nutrition, Federal University of Bahia (ENUFBA) (reference number 67205423.6.0000.5023). This study waived informed consent, because this study uses electronic data without any personally identifiable information.

## Results

During the study period, 3,801,158 children younger than 5 y and 16,483,960 observations were recorded in this study. After applying the exclusion criteria, we retained 2,021,998 (53.2%) children with 2 or more repeated measurements and 8,726,599 observations (Figure 1).

Overall, 7.0% of all live births included in the study were preterm, 7.2% were LBW, and 9.8% were SGA. Of these, 16.7% were SVNs. Among preterm births, the preterm + AGA + NBW phenotype was the most prevalent, accounting for ∼50% of preterm births and 3.6% of all live births. Preterm + AGA + LBW and preterm + SGA + LBW comprised 2.8% and 0.6 % of all births, respectively. Among term births, the term + AGA + NBW phenotype was the most prevalent, representing 90% of term births and 83.4% of all live births. Term + SGA + NBW, term + SGA + LBW, and term + AGA + LBW accounted for 5.8%, 3.5%, and 0.4% of all births, respectively (Figure 1). The distribution of measurements by gender and vulnerability phenotype can be seen in Supplemental Figure 1. The distribution of measurements at each knot evaluated in the analyses is also available in Supplemental Figure 2. The prevalence of small vulnerable births was higher among mothers who were single/widow/divorced (47.6%), maternal education (<3 y 9.3%; 4–7 y 34.4%), younger than 20 y (25.9%), and >35 y of age (9.9%), nulliparous (34.8%), underwent fewer antenatal visits (1–3 11.0%; 4–6 visits 37.5%), and had a vaginal delivery (59.2%) compared with not small vulnerable births (Table 1). The characteristics of each vulnerability phenotype are shown in Supplemental Table 1.

**TABLE 1:** Characteristics of live births by vulnerability status in Brazil from 2011 to 2017 (*n* = 2,021,998)

**Variables**	Total births	Not small vulnerable newborns^1^	Small vulnerable newborns^2^	*P* value^3^
	2,021,998 (100%)	1,685,326 (83.3%)	336,672 (16.7%)	
	*N* (%)	*N* (%)	*N* (%)	
Residence region				
North	278,009 (13.8)	232,454 (13.8)	45,555 (13.5)	0.000
Northeast	890,308 (44.0)	747,729 (44.4)	142,579(42.4)	
Southeast	525,867 (26.0)	432,358 (25.7)	93,509 (27.8)	
South	200,117 (9.9)	165,619 (9.9)	34,498 (10.3)	
Midwest	127,697 (6.3)	107,166 (6.4)	20,531 (6.1)	
Missing^4^	0 (0.0)			
Residence area				
Urban	1,456,658 (72.0)	1,213,012(72.0)	243,646 (72.4)	0.000
Rural	565,239 (28.0)	472,240 (28.0)	92,999 (27.6)	
Missing^4^	101 (0.0)			
Household overcrowding (inhabitants per room)				
<2	1,800,935 (94.8)	1,502,113 (94.9)	298,822 (94.5)	0.000
≥2	98,663 (5.2)	81,360 (5.1)	17,303 (5.5)	
Missing^4^	122,400 (6.0)			
Marital status				
Married/civil partnership	1,085,684 (54.5)	911,690 (54.9)	173,994 (52.4)	0.000
Single/widow/divorced	907,597 (45.5)	749,759 (45.1)	157,838 (47.6)	
Missing^4^	28,717 (1.4)			
Maternal education (y)				
<3	166,125 (8.4)	135,427 (8.2)	30,724 (9.3)	0.000
4–7	653,421 (33.0)	540,055 (32.7)	113,366 (34.4)	
8–11	1,113,079 (56.2)	934,771 (56.7)	178,308 (54.1)	
≥12	47,125 (2.4)	39,936 (2.4)	7,189 (2.9)	
Missing^4^	42,222 (2.1)			
Maternal race				
White	427,515 (22.0)	356,524 (22.0)	70,991 (22.0)	0.000
Mixed race	1,365,169 (70.4)	1,140,727 (70.5)	224,442 (69.7)	
Black	125,912 (6.5)	103,174 (6.4)	22,738 (7.1)	
Indigenous	21,530 (1.1)	17,547 (1.1)	3,983 (1.2)	
Asian	5,528 (0.28)	4,577 (0.28)	951 (0.29)	
Missing^4^	76,344 (3.8)			
Maternal age (y)				
<20	451,325 (22.3)	364,094 (21.6)	87,231 (25.9)	0.000
20–34	1,39,680 (60.0)	1,178,717 (69.9)	215,963 (64.2)	
≥35	175,990 (8.7)	142,513 (8.5)	33,477 (9.9)	
Missing	3 (0.0)			
Number of previous pregnancies				
None	561,638 (29.5)	452,238 (28.4)	103,400 (34.8)	0.000
1–3	1,122,633 (58.9)	955,509 (60.0)	167,124 (53.1)	
4+	222,286 (11.7)	184,048 (11.6)	38,238 (12.2)	
Missing^4^	115,441(5.7)			
Number of prenatal visits				
None	33,879 (1.7)	26,230 (1.6)	7,649 (2.3)	0.000
1–3	153,605 (7.6)	116,705 (7.0)	36,900 (11.0)	
4–6	630,702 (31.8)	515,366 (30.7)	125,336 (37.5)	
7+	1,183,265 (58.8)	1,018,935(60.8)	164,330 (49.2)	
Missing^4^	10,547 (0.5)			
Type of delivery				
Vaginal	1,161,821 (57.6)	962,801 (57.2)	199,020 (59.2)	0.000
Cesarean section	856,391 (42.4)	719,397 (42.8)	136,994 (40.8)	
Missing^4^	3,786 (0.2)			
Gender of newborn				
Male	1,018,971 (50.4)	847,877 (50.3)	171,094 (50.8)	0.000
Female	1,003,027 (49.6)	837,449 (49.7)	165,578 (49.2)	
Missing^4^	0 (0.0)			

1 Not small vulnerable newborns = term + AGA + NBW (term + adequate-for-gestational age + normal birth weight).

2 Small vulnerable newborns (those with at least one of the phenotypes, i.e., preterm birth, SGA, and LBW).

3 Variables were analyzed by *χ*^2^ tests.

4 Percentage was not included when calculating the categories.

**FIGURE 2:**
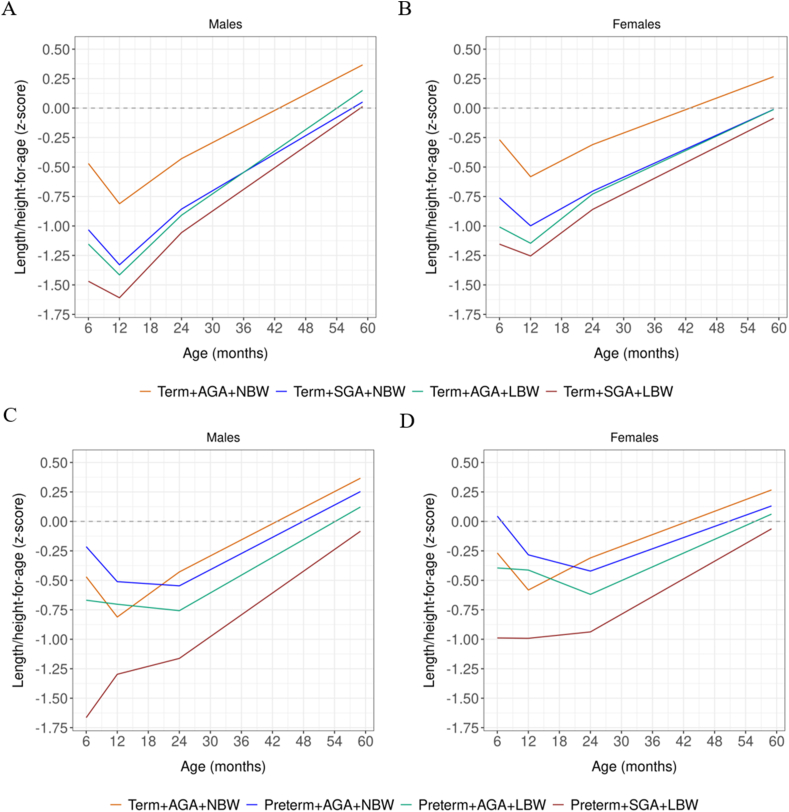
Length/height-for-age growth trajectories (*z*-score) by vulnerability phenotype and gender from 6 to 59 mo postnatal age. (A) Growth trajectory for males (term); (B) growth trajectory for females (term); (C) growth trajectory for males (term and preterm); (D) growth trajectory for females (term and preterm)**;** SD 0.0 = median World Health Organization.

The L/HAZ growth trajectories for all term phenotypes exhibited a reduction between 6 and 12 mo, followed by a period of growth recovery from 12 mo onward. However, these trajectories appeared to be lower among term + SGA + LBW children (Figure 2A, B). In the case of preterm phenotypes, we observed a reduction in L/HAZ growth trajectories between 6 and 24 mo, with a delayed onset of growth recovery between 24 and 59 mo. Children with preterm phenotypes seemed to be shorter until 5 y compared with those born at term. This condition was particularly pronounced among preterm + SGA + LBW children (Figure 2C, D).

**FIGURE 3:**
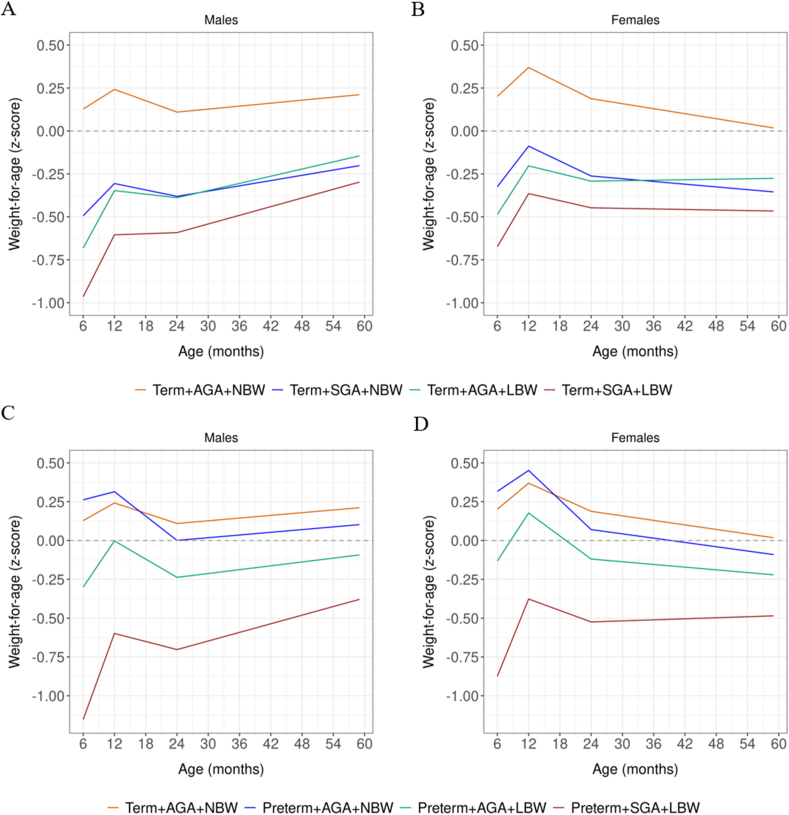
Weight-for-age growth trajectories (*z*-score) by vulnerability phenotype and gender from 6 to 59 mo postnatal age. (A) Growth trajectory for males (term); (B) growth trajectory for females (term); (C) growth trajectory for males (preterm); (D) growth trajectory for females (preterm); SD 0.0 = median World Health Organization.

Regardless of phenotype, we observed an increase in WAZ growth trajectories for males between 6 and 12 mo, followed by a decrease from 12 to 24 mo and a subsequent weight regain from 24 mo (Figure 3A, C). The trajectories appear to be similar for females ≤24 mo, after which they continue a downward trajectory (Figure 3B, D). Notably, at 12 mo, the growth trajectories for preterm + AGA + NBW were slightly higher compared with those for term + AGA + NBW in both genders (Figure 3C, D). The mean L/HAZ and WAZ by gender and vulnerability phenotype are shown in Supplemental Table 2.

With few differences, females seemed to follow the same growth pattern observed among males. However, the average L/HAZ and WAZ for females were higher than those for males in the first 24 mo of postnatal life. This situation was reversed between 24 and 59 mo, with females showing lower mean L/HAZ than males (FIGURE 2, FIGURE 3B, D and Supplemental Table 2).

Growth velocities indicated that those born at term initiate the L/HAZ growth recovery period earlier, at 12–24 mo for all phenotype groups. Among male term children, catch-up growth was observed at 24–59 mo: term + AGA + NBW (Δ = 0.80), term + SGA + NBW (Δ = 0.91), term + AGA + LBW (Δ = 1.06), and term + SGA + LBW (Δ = 1.07). In contrast, preterm males and all females seemed to have slower growth recovery compared with their term-born and male counterparts, respectively. However, catch-up growth was also evident at 24–59 mo for males preterm: preterm + AGA + NBW (Δ = 0.80), preterm + AGA + LBW (Δ = 0.88), and preterm + SGA + LBW (Δ = 1.08); and among females: term + SGA + NBW (Δ = 0.69), term + AGA + LBW (Δ = 0.72), term + SGA + LBW (Δ = 0.77), preterm + AGA + LBW (Δ = 0.68), and preterm + SGA + LBW (Δ = 0.83). The changes in WAZ did not exceed the predefined threshold (>0.67) (Table 2).

**TABLE 2:** Change of *z*-scores (growth velocities) for length/height-for-age and weight-for-age by vulnerability phenotype and gender, 2011–2017 (*n* = 2,021,998)

Vulnerability phenotype	Males		Females	
	Length/height-for-age	Weight-for-age	Length/height-for-age	Weight-for-age
	Δ^1^	Δ^1^	Δ^1^	Δ^1^
Term + AGA + NBW (mo)				
6–12	−0.34	0.11	−0.31	0.17
12–24 mo	0.38	−0.13	0.27	−0.18
24–59 mo	0.80^2^	0.10	0.58	−0.17
Term + SGA + NBW (mo)				
6–12	−0.30	0.19	−0.24	0.24
12–24	0.47	−0.07	0.29	−0.17
24–59	0.91^2^	0.18	0.69^2^	−0.09
Preterm + AGA + NBW (mo)				
6–12	−0.30	0.05	−0.33	0.13
12–24	−0.04	−0.31	−0.14	−0.38
24–59	0.80^2^	0.10	0.55	−0.16
Term + AGA + LBW (mo)				
6–12	−0.26	0.33	−0.14	0.28
12–24	0.51	−0.04	0.42	−0.09
24–59	1.06^2^	0.24	0.72^2^	0.02
Term + SGA + LBW (mo)				
6–12	−0.14	0.36	−0.10	0.31
12–24	0.55	0.01	0.39	−0.08
24–59	1.07^2^	0.29	0.77^2^	−0.02
Preterm + AGA + LBW (mo)				
6–12	−0.03	0.30	−0.02	0.31
12–24	−0.06	−0.24	−0.21	−0.30
24–59	0.88^2^	0.14	0.68^2^	−0.10
Preterm + SGA + LBW (mo)				
6–12	0.37	0.55	0.00	0.50
12–24	0.13	−0.10	0.05	−0.15
24–59	1.08^2^	0.32	0.87^2^	0.04

1 Change (Δ) in the mean *z*-score between 2 time points.

2 Catch-up growth − change in *z*-score > 0.67.

In additional analyses, we observed an increase in WLZ/WHZ growth trajectories for all phenotypes between 6 and 12 mo, followed by a decrease from 12 to 24 mo. From 24 to 59 mo, children continued with a downward trajectory. These trajectories appeared to be thinner among the most vulnerable children, especially in the term + SGA + LBW and preterm + SGA + LBW phenotypes (Supplemental Figure 3). Also, the trajectories appeared to be similar between genders. Regarding growth velocities, no catch-up was observed for any of the vulnerability profiles (Supplemental Table 3).

Scatter plots of observed compared with predicted values, visually representing the model’s prediction accuracy, suggest a good fit between observed and adjusted data (Supplemental Figures 4–7). The percentage of explained variation (*R*-squared) for WAZ and L/HAZ was >80% for all vulnerability phenotypes (Supplemental Table 2).

In additional analyses that retained the records with negative heights (Supplemental Figure 8), the postnatal growth trajectories of SVNs were consistent with the results of our initial analyses (Supplemental Figures 9 and 10). However, by the end of the follow-up, we observed that the mean L/HAZ and WAZ were below the WHO reference standard median for all vulnerability phenotypes (Supplemental Table 4). In addition, no catch-up growth was observed (Supplemental Table 5).

## Discussion

In this study, we evaluated the growth trajectory of >2 million children during the first 5 y of life, including 336,672 (16.7%) live births with at least one of the vulnerable phenotypes, of which 11,350 (0.6%) had simultaneously preterm, LBW, and SGA. Children born preterm seem to remain shorter and thinner during childhood than those born at term, a condition aggravated among the most vulnerable (simultaneously preterm, LBW, and SGA). The height catch-up growth occurred at 24–59 mo for males of all groups (term and preterm). For females, the catch-up growth occurred only for term + SGA + NBW, term + AGA + LBW, term + SGA + LBW, preterm + AGA + LBW, and preterm + SGA + LBW.

Low- and middle-income countries (LMICs) continue to bear a significant burden of stunting [47]. In Brazil, in 2017, a high prevalence of stunting (12.4%) and wasting (5.1%) was observed among children monitored in the Unified System of Health (SUS)’s primary health services [48]. These burdens may be even higher when considering the combinations of preterm, SGA, and LBW categories. Christian et al. [47] found that SGA and full-term birth were associated with a 2.4 higher chance of stunting compared with AGA and full-term, and AGA and preterm with a 1.9 greater chance. Furthermore, the odds ratio increased to 4.5 for SGA and preterm births. Similar associations were also observed for wasting [47].

Studies in LMICs using longitudinal data and large samples to evaluate child growth are scarce [[49], [50], [51]]. Anthropometric measurements provide greater consistency when measured repeatedly over a given period than cross-sectional or 1-point measurements [52]. Additionally, examining growth trajectories compared with dichotomous outcomes (e.g., stunting or wasting) has advantages, such as the longitudinal assessment of growth and capturing small variations in anthropometric measurements [53]. Furthermore, to the best of our knowledge, no studies evaluated growth trajectories according to different phenotypical conditions at birth, as proposed by Ashorn et al. [10], which makes our study unique. Therefore, understanding these growth trajectories can help identify infants at high risk of growth impairment and thus support better neonatal management, considering that there are many health problems related to childhood inadequate growth trajectory [54].

The evidence presented here on child growth was consistent with the literature, showing that preterm, SGA, and LBW, isolated, or preterm in combination with SGA children remain relatively lighter and shorter than their peers during childhood [[55], [56], [57], [58], [59]]. Studies have shown that preterm children grow below reference standards and rarely reach the growth of full-term children in the first years of life, a condition that worsens when associated with SGA at birth [49,60,61]. Consistent with previous studies in LMICs, our L/HAZ growth trajectories support the observation that males are born less adequate for height than females, and both genders remain below the median of international gender- and age-specific references throughout the first 1000 d [49,62].

In addition, studies have reported that children born very prematurely and SGA may be less likely to catch-up growth during early childhood [[63], [64], [65]], and their height recovery may be delayed beyond 6 y of age [64]. Raaijmakers et al. [63] revealed that extremely LBW preterm infants failed to thrive during the first 2 y of life and found a positive change in height and weight *z*-score from 24 mo – a result similar to what we found [63].

Although we observed a catch-up in L/HAZ among vulnerable children, no catch-up growth was noted for WAZ and WLZ/WHZ. The growth trajectories and velocities of WLZ/WHZ indicated a gradual and progressive decrease between 24 and 59 mo for all phenotypes, including term + AGA + NBW. This growth pattern is consistent with BMI trajectories observed in previous studies [66,67]. Generally, BMI rapidly increases during the first year of life, then subsequently decreases and reaches a nadir around 6–7 y of age. However, the WLZ/WHZ trajectories tended to be more aggravated among females and SVN phenotypes, making these groups more vulnerable to moderate and severe wasting and, consequently, elevated risk of death [68].

In developing countries, the catch-up growth of small children born biologically vulnerable may be compromised, probably due to unfavorable socioeconomic conditions. Thus, the great concern is that these children do not reach their optimum growth potential [49,51,60]. The fact is that, in LMIC, numerous factors, including higher levels of inadequate lactation and infant and young child-feeding practices, infections, difficult access to health services, and other environmental exposures undermine children’s ability to grow and thrive healthily [47], especially among SNV who are at increased risk of several adverse health outcomes during childhood, including stunted growth, noncommunicable diseases, long-term disability, and reduced learning potential [13].

Victora and Barros coined the phrase "the catch-up dilemma" to describe the benefits and drawbacks of catch-up growth on small infants’ health in the short and long term [69]. Early growth (fast growth in infancy among small newborns) is advantageous for better neurodevelopmental outcomes, lower risk of hospitalizations, mortality, and persistent short stature [[70], [71], [72], [73]]. However, research over many decades has shown associations between early catch-up and risk of cardiovascular and metabolic diseases, including overweight and obesity, in late childhood and adulthood [74,75]. Our results showed that preterm and small children experience growth recovery later than those born at term and NBW. Despite this piece of evidence, the long-term effects of delayed recovery are unclear, and future study is needed to answer this question. Thus, it is emphasized that monitoring the growth trajectory in all children is an essential part of child health care, especially for the most vulnerable infants.

Health promotion, prevention, and assistance actions targeting pregnant women and newborns directly influence their health condition throughout childhood and into adulthood [76]. In Brazil, various programs, policies, and strategies have been created and implemented in health services to enhance child health care [77]. Within this framework, the Ministry of Health launched the Rede Cegonha in 2011 – an innovative strategy focused on the organization and implementation of actions for the health care of children aged 0–24 mo, with the goal of ensuring their healthy growth and development [78]. Hence, there is a crucial need for systematic monitoring of child growth and associated risk factors, facilitating the early detection of modifiable changes.

### Strengths and limitations

The present study has strengths and limitations. This population-based longitudinal study has a sample size with sufficient power to assess growth trajectories for different vulnerability phenotypes, even for less-prevalent phenotypes. Additionally, we used a longitudinal design to investigate child growth trajectories rather than a dichotomous approach, which allowed us to assess changes in trajectories over time [53]. Another important strength is the use of corrected postnatal age to assess standardized anthropometric measurements, considering variations in newborn size due to the heterogeneity of gestational age at birth. In addition, the Broken-stick model is recommended for assessing irregular individual trajectories and standardized *z*-score data, providing easily interpretable estimates of childhood growth trajectories [42,43].

However, some limitations are noted. First, the use of secondary data, which was not designed primarily for research purposes, may be susceptible to some limitations related to missing, underestimation, and potential misclassification. We limited our analyses to children older than 6 mo because, to date, we do not have a single reference standard or compatible references that can assess the growth trajectories of preterm children from birth to 5 y of age. Thus, future studies are essential to improve our understanding of growth trajectories from birth to 6 mo in the studied population. The strategy of excluding inconsistencies in the height variable (negative difference between 2 subsequent ordered measurements) may introduce bias into our results, potentially leading to higher growth velocities. Therefore, a cautious interpretation is necessary. Known errors and limitations related to the collection and entry of anthropometric measurements into SISVAN add to these considerations [29]. For instance, the data quality is anticipated to be poorer, particularly in the group of children under 2 y of age, whose length/height is measured with the child lying down [35]. However, we observed in the sensitivity analysis, which maintains negative heights in the data set, that the postnatal growth trajectories for the SVNs were consistent with the results of our initial analyses. The Broken-stick is a univariate model and does not allow the inclusion of covariates. Consequently, data on maternal and neonatal diseases, lactation, and socioeconomic conditions – factors that may influence growth trajectories – were not considered. Therefore, the results of our study must be interpreted with caution. In addition, this model shows better convergence when fitted to larger data sets [42]. Finally, this study was carried out among the poorest population of an upper middle-income country with a history of great social and health inequalities [79], so the results of our study may be more generalizable for children born in similar conditions.

In conclusion, children born at term appear to show the recovery of WAZ and LAZ/HAZ earlier than preterm. Despite experiencing a recovery of growth, children born preterm seem to remain shorter and thinner throughout childhood compared with those born at term. This condition is exacerbated among the most vulnerable children, particularly those with preterm + SGA + LBW phenotype. A comprehensive understanding of postnatal growth is critical to improving long-term outcomes in newborns. Hence, the results of our study can contribute to strengthening public policies and developing nutritional strategies focused on the weight and height recovery of SVNs. This involves enhancing child growth monitoring systems, optimizing interventions, and efficiently allocating resources. Such efforts hold promise for advancing the achievement of the global nutrition targets and the SDGs related to ending all forms of malnutrition.

## Acknowledgments

We thank the CIDACS/FIOCRUZ data production team’s staff for their work on linking the data used in this study, and providing information on data quality. We would also like to thank the IT team for their efforts in helping to facilitate our access to data. We also thank Nandita Perumal for the contributions to the critical revision of the manuscript.

## Author contributions

The authors’ responsibilities were as follows – ASR, RCRS: conceptualized and designed the study, drafted and revised the manuscript; ASR, JFMS, EJP, NJS: contributed to statistical analysis; ESP, GK, RLF, CA: contributed to data interpretation, and critically reviewed the intellectual content of the manuscript; RCRS, MLB, LCR: acquired data, contributed to data interpretation, and critically reviewed the intellectual content of the manuscript; and all of the authors approved the final, submitted version of this manuscript, and accepted accountability for all aspects of this work.

## Conflict of interests

We declare no competing interests

## Funding

This study was funded by the Department of Science and Technology (Decit), Ministry of Health (Decentralized Execution Term – TED number 64/2022, process 415 25000.148278/2022-10). CIDACS and the 100 Million cohort received financial support from the Wellcome Trust (grant number 202912/Z/16/Z), the Health Surveillance Secretariat, Ministry of Health, Brazil, Bahia State (TED number 159/2019), the Secretariat of Science and Technology of the State of Bahia (SECTI) (term of assignment of movable property 048/2018, process number 1430150022698). ESP is a fellow supported by the Wellcome Trust (grant number 225925/Z/22/Z). NJS acknowledges support from the grant CEX2018-000806-S funded by MCIN/AEI/ 10.13039/501100011033 and support from the Generalitat de Catalunya through the CERCA Program. The funders had no role in study design, data collection, and analysis, and on the decision to publish or preparation of the manuscript. The authors received no specific funding for this work.

## Data availability

All data supporting this study were obtained from the Center for Data and Knowledge Integration for Health (CIDACS). These were licensed for exclusive use in the present study and, due to the privacy rules of the Brazilian Laws and Ethics Committee, are not openly available. Upon request with adequate justification and approval of an ethics committee, controlled access to data is considered and, if possible, allowed access. The data described in the manuscript, code book, and analytical code will be made available upon request to the corresponding author, E-mail: aline.srocha@fiocruz.br. To access the data, each researcher should present a research project, ethical approval, and a data plan, to extract an unidentified/anonymized data set for analysis. Further information can be obtained at https://cidacs.bahia.fiocruz.br/acesso-aos-dados/.
