# Supplementary material for: Postnatal growth in small vulnerable newborns: a longitudinal study of 2 million Brazilians using routine register-based linked data
Source: Am J Clin Nutr. 2023 Dec 20;119(2):444–55. doi: 10.1016/j.ajcnut.2023.12.009 (PMC10884605; doi:10.1016/j.ajcnut.2023.12.009)
Supplement: Multimedia component 1 [file mmc1.docx]

**Supplementary Materials**

**Supplemental Table 1**: Characteristics of live births included in the study by vulnerability phenotype in Brazil from 2011-2017, (n= 2,021,998)

| Variables | Term+AGA+NBW | Term+SGA+NBW | Preterm+AGA+NBW | Term+AGA+LBW | Term+SGA+LBW | Preterm+AGA+LBW | Preterm+SGA+LBW |
| --- | --- | --- | --- | --- | --- | --- | --- |
|  | N % | N% | N (%) | N (%) | N (%) | N (%) | N (%) |
|  | 11,685,326 (83.4%) | 117,253 (5.8%) | 73,031 (3.6%) | 8,524 (0.4%) | 69,940 (3.5%) | 56,564 (2.8%) | 11,360 (0.6%) |
| Residence region | |  |  |  |  |  |  |
| North | 232,454 (13.8) | 17,315 (14.8) | 10,773 (14.8) | 916 (10.8) | 9,238 (13.2) | 6,171 (10.9) | 1,142 (10.0) |
| Northeast | 747,729 (44.4) | 52,212 (44.5) | 30,312(42.2) | 3,248 (38.1) | 29,606 (42.4) | 22,190 (39.2) | 4,511 (39.7) |
| Southeast | 432,358 (25.7) | 30,113 (25.7) | 19,593 (26.8) | 2,730 (32.0) | 19,531 (27.9) | 17,808 (31.5) | 3,734 (32.9) |
| South | 165,619 (9.8) | 10,585 (9.0) | 7,273 (10.0) | 1,083 (12.7) | 7,293 (10.4) | 6,957 (12.3) | 1,307 (11.5) |
| Central-west | 107,166 (6.3) | 7,028 (6.0) | 4,580 (6.2) | 547 (6.4) | 4,272 (6.1) | 3,438 (6.1) | 666 (5.9) |
| Residence area | |  |  |  |  |  |  |
| Urban | 1,213,012 (72.0) | 81,293 (69.3) | 53,232 (72.9) | 6,568 (77.1) | 50,290 (71.9) | 43,511 (76.9) | 8,752 (77.0) |
| Rural | 472,240 (28.0) | 35,956 (30.7) | 19,795 (27.1) | 1,956 (22.9) | 19,639 (28.1) | 13,045 (23.1) | 2,608 (23.00) |
| Household overcrowding | | |  |  |  |  |  |
| <2 inhabitants per room | 1,502,113 (94.9) | 103,370 (94.3) | 64,575 (94.2) | 7,691 (95.4) | 62,030 (94.6) | 50,847 (95.2) | 10,309 (95.7) |
| ≥2 inhabitants per room | 81,360 (5.1) | 6,302 (5.7) | 4,015 (5.8) | 370 (4.6) | 3,568 (5.4) | 2,580 (4.8) | 468 (4.3) |
| Marital status |  |  |  |  |  |  |  |
| Married/ Civil partnership | 911,690 (54.9) | 60,900 (52.8) | 38,378 (53.3) | 4,412 (52.4) | 35,962 (52.2) | 28,461 (50.9) | 5,881 (52.5) |
| Single/widow/divorced | 749,759 (45.1) | 54,8537 (47.2) | 33,565 (46.7) | 4,014 (47.6) | 32,955 (47.8) | 27,439 (49.1) | 5,328 (47.5) |
| Maternal education (years) | | | | | | | |
| < 3 | 135,427 (8.2) | 10,937 (9.5) | 6,688 (9.4) | 641 (7.7) | 7,111 (10.4) | 4,349 (7.8) | 998 (9.0) |
| 4-7 | 540,055 (32.7) | 40,508 (35.3) | 24,190 (33.9) | 2,855 (34.1) | 23,898 (34.9) | 18,389 (33.1) | 3,526 (31.7) |
| 8-12 | 934,771 (56.7) | 61,054 (53.3) | 38,876 (54.4) | 4,660 (55.7) | 35,992 (52.6) | 31,457 (56.6) | 6,269 (56.3) |
| 12 + | 39,936 (2.4) | 2,145 (1.9) | 1,669 (2.3) | 214 (2.5) | 1,440 (2.1) | 1,379 (2.5) | 342 (3.1) |
| Maternal race/ethnicity | | | | | | | |
| White | 356,524 (22.0) | 22,235 (19.8) | 15,889 (22.7) | 2,044 (25.0) | 14,430 (21.5) | 13,698 (25.4) | 2,695 (25.0) |
| Mixed-race | 1,140,727 (70.5) | 80,980 (72.0) | 48,347 (69.1) | 5,467 (66.8) | 46,689 (69.7) | 35,808 (66.5) | 7,151 (66.3) |
| Black | 103,174 (6.4) | 7,754 (6.9) | 4,582 (6.6) | 603 (7.4) | 5,054 (7.6) | 3,883 (7.2) | 862 (8.0) |
| Indigenous | 17,547 (1.1) | 1,458 (1.3) | 1,126 (1.6) | 69 (0.8) | 808 (1.2) | 444 (0.8) | 78 (0.7) |
| Asian | 4,557 (0.3) | 323 (0.29) | 202 (0.3) | 25 (0.3) | 168 (0.3) | 168 (0.3) | 37 (0.3) |
| Maternal age (years) | | | | | | | |
| 14-19 | 364,094 (21.6) | 31,124 (26.5) | 19,317 (26.4) | 2,190 (25.7) | 17,081 (24.4) | 15,170 (26.8) | 2,349 (20.6) |
| 20-34 | 1,178,717 (70.0) | 76,924 (65.6) | 46,493 (63.7) | 5,382 (63.1) | 45,161 (64.6) | 34,678 (61.3) | 7,325 (64.6) |
| 35-49 | 142,513 (8.4) | 9,205 (7.9) | 7,220 (9.9) | 952 (11.2) | 7,698 (11.0) | 6,716 (11.9) | 1,686 (14.8) |
| Number of previous pregnancies | | | | | | | |
| None | 452,238 (28.4) | 39,145 (36.1) | 21,634 (31.4) | 2,823 (35.1) | 23,142 (35.5) | 18,967 (35.5) | 3,689 (34.3) |
| 1 a 3 | 955,509 (60.0) | 57,091 (52.6) | 38,630 (56.1) | 4,233 (52.7) | 33,925 (52.0) | 27,636 (51.7) | 5,609 (52.2) |
| 4+ | 184,048 (11.6) | 12,261 (11.3) | 8,573 (12.5) | 978 (12.2) | 8,169 (12.5) | 6,808 (12.8) | 1,449 (13.5) |
| Number of prenatal visits | | | | | | | |
| None | 26,230 (1.6) | 2,234 (1.91) | 1,352 (1.9) | 198 (2.4) | 1,656 (2.4) | 1,786 (3.2) | 423 (3.8) |
| 1 a 3 | 116,705 (7.0) | 9,217 (7.90) | 8,957 (12.3) | 85 (10.2) | 6,739 (9.7) | 9,514 (17.0) | 1,608 (14.3) |
| 4 a 6 | 515,366 (30.7) | 36,072 (30.9) | 30,538 (42.1) | 3,166 (37.4) | 23,909 (34.4) | 26,607 (47.6) | 5,044 (45.0) |
| 7+ | 1,018,935 (60.7) | 69,138 (59.7) | 31,734 (43.7) | 4,234 (50.0) | 37,088 (53.5) | 17,998 (32.2) | 4,138 (36.9) |
| Type of delivery | | | | | | | |
| Vaginal | 962,801 (57.2) | 73,382 (62.7) | 44,492 (61.0) | 5,023 (59.0) | 41,157 (59.0) | 30,497 (54.0) | 4,467 (39.4) |
| Cesarean section | 719,397 (42.8) | 43,629 (37.3) | 28,405 (39.0) | 3,488 (41.0) | 28,638 (41.0) | 25,962 (46.0) | 6,872 (60.6) |
| Newborns` sex | | | | | | | |
| Male | 847,877 (50.3) | 63,725 (54.3) | 41,806 (57.2) | 3,123 (36.6) | 29,030 (41.5) | 27,914 (49.4) | 5,496 (48.4) |
| Female | 837,449 (49.7) | 53,528 (45.7) | 31,225 (42.8) | 5,401 (63.4) | 40,910 (58.5) | 28,650 (50.6) | 5,864 (51.6) |


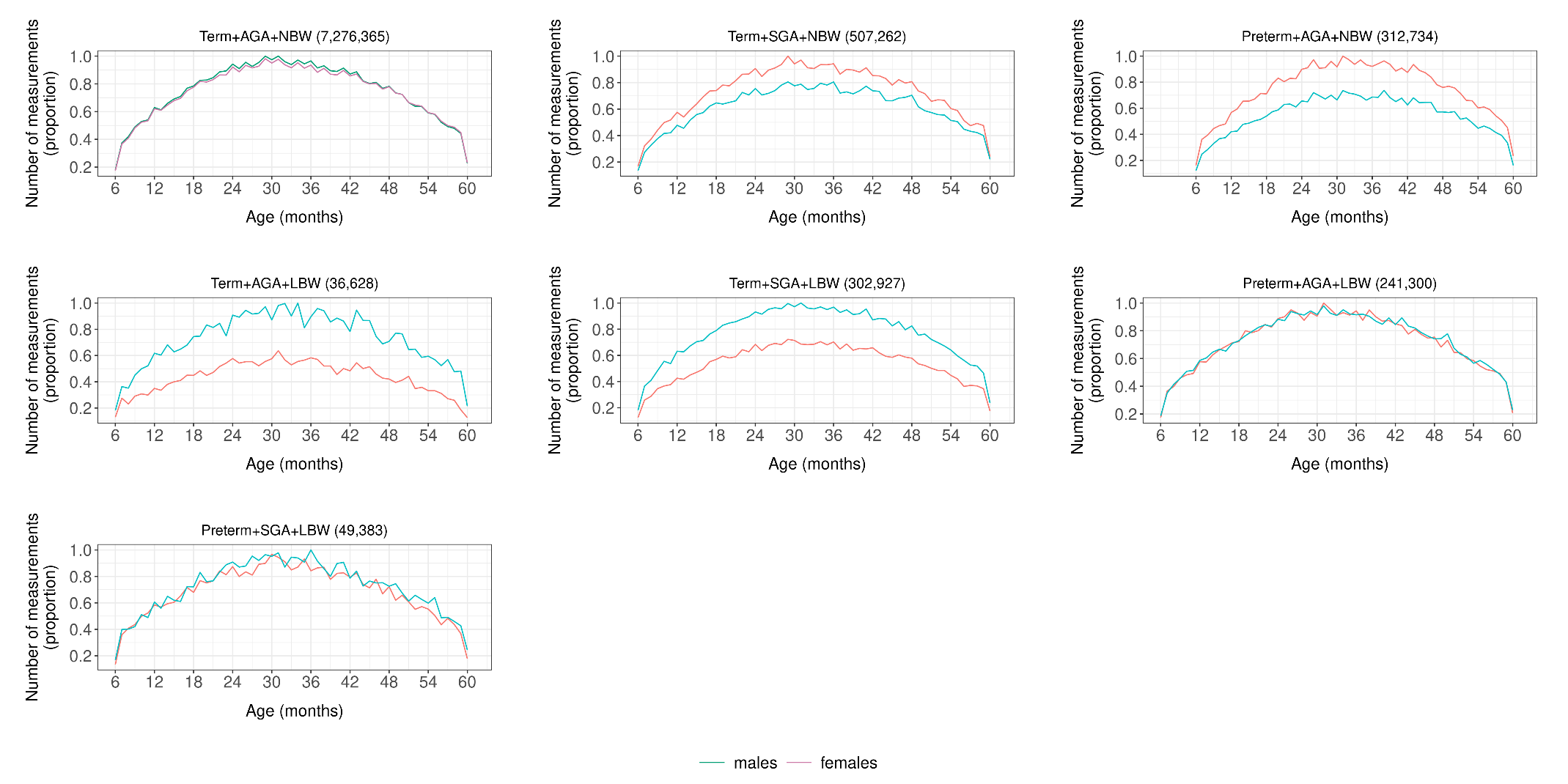


**Supplemental Figure 1**: Distribution of measurements by sex of vulnerability phenotype, 2011-2017 (n= 2,021,998)


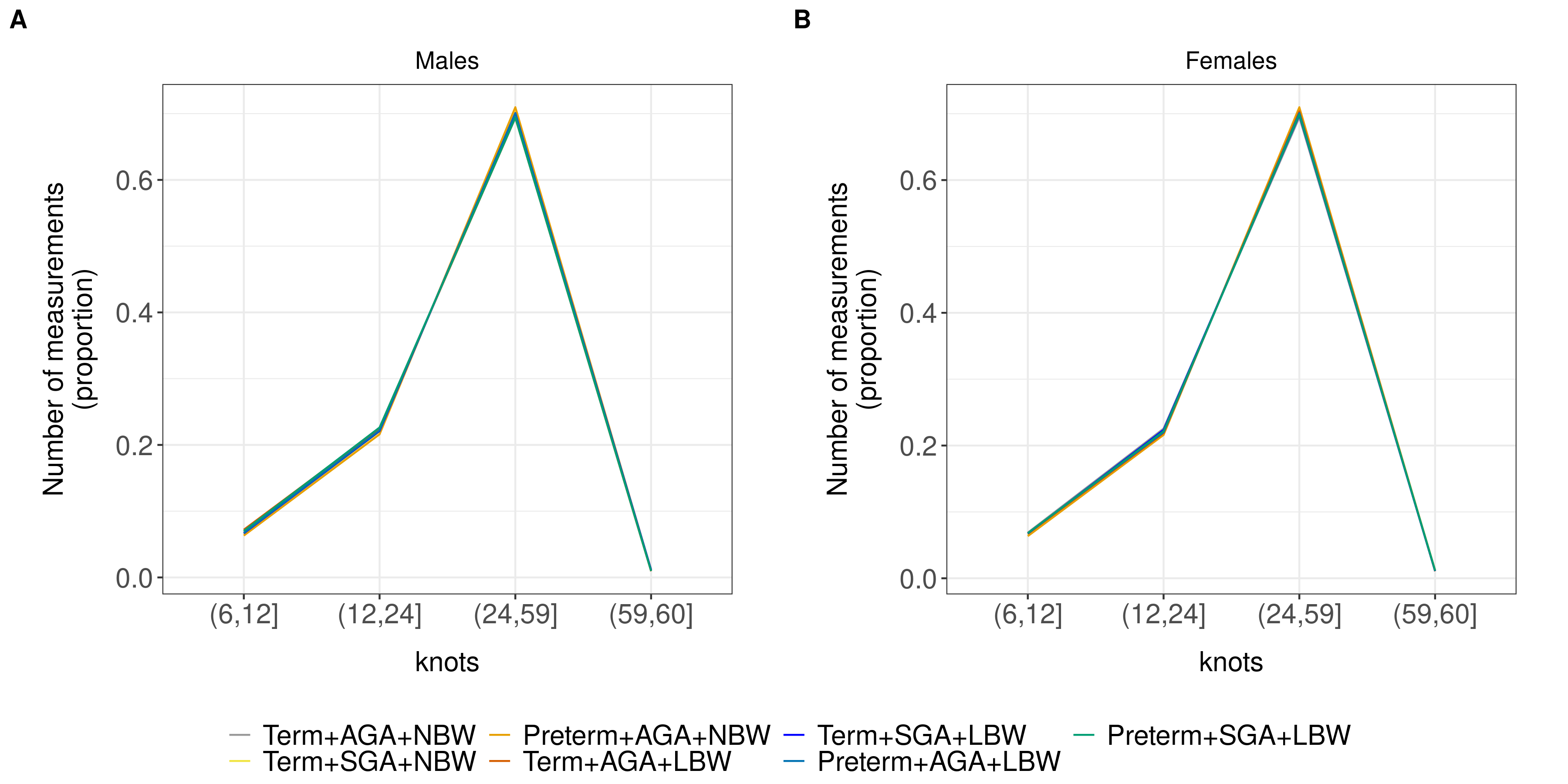


**Supplemental Figure 2**: Distribution of measurements at each knot by sex of vulnerability phenotype, 2011-2017 (n= 2,021,998)

| Supplemental Table 2: Longitudinal anthropometric indicators (z-scores) by vulnerability phenotype and sex, 2011-2017 (n= 2,021,998) | | | | | | | | |
| --- | --- | --- | --- | --- | --- | --- | --- | --- |
| Vulnerability  phenotype | **Males** | | | | **Females** | | | |
|  | Length/height-for-age | | Weight-for-age | | Length/height-for-age | | Weight-for-age | |
|  | mean | SD^1^ | mean | SD^1^ | mean | SD^1^ | mean | SD^1^ |
| Term+AGA+NBW | | | | | | | | |
| Month 6 | -0.47 | 1.90 | 0.13 | 1.12 | -0.27 | 1.76 | 0.20 | 1.04 |
| Month 12 | -0.81 | 1.99 | 0.24 | 1.13 | -0.58 | 1.86 | 0.37 | 1.03 |
| Month 24 | -0.43 | 1.59 | 0.11 | 1.14 | -0.31 | 1.55 | 0.19 | 1.10 |
| Month 59 | 0.37 | 1.59 | 0.21 | 1.31 | 0.27 | 1.58 | 0.02 | 1.25 |
| R^2^ | 0.84 | | 0.84 | | 0.83 | | 0.84 | |
| Term+SGA+NBW | | | | | | | | |
| Month 6 | -1.03 | 1.87 | -0.49 | 1.10 | -0.76 | 1.76 | -0.32 | 1.02 |
| Month 12 | -1.33 | 2.00 | -0.31 | 1.13 | -1.00 | 1.92 | -0.09 | 1.07 |
| Month 24 | -0.86 | 1.63 | -0.38 | 1.14 | -0.70 | 1.59 | -0.26 | 1.12 |
| Month 59 | 0.05 | 1.67 | -0.20 | 1.33 | -0.01 | 1.65 | -0.35 | 1.28 |
| R^2^ | 0.84 | | 0.83 | | 0.83 | | 0.83 | |
| Preterm+AGA+NBW | | | | | | | | |
| Month 6 | -0.21 | 1.99 | 0.26 | 1.18 | 0.04 | 1.80 | 0.32 | 1.03 |
| Month 12 | -0.51 | 2.04 | 0.31 | 1.17 | -0.28 | 1.94 | 0.45 | 1.06 |
| Month 24 | -0.55 | 1.62 | 0.00 | 1.16 | -0.42 | 1.58 | 0.07 | 1.14 |
| Month 59 | 0.25 | 1.62 | 0.10 | 1.36 | 0.13 | 1.58 | -0.09 | 1.29 |
| R^2^ | 0.84 | | 0.85 | | 0.84 | | 0.84 | |
| Term+AGA+LBW | | | | | | | | |
| Month 6 | -1.15 | 1.74 | -0.68 | 1.17 | -1.01 | 1.61 | -0.49 | 1.01 |
| Month 12 | -1.42 | 2.05 | -0.35 | 1.14 | -1.15 | 1.83 | -0.20 | 1.09 |
| Month 24 | -0.91 | 1.55 | -0.39 | 1.13 | -0.73 | 1.58 | -0.29 | 1.12 |
| Month 59 | 0.15 | 1.70 | -0.14 | 1.44 | -0.01 | 1.60 | -0.28 | 1.31 |
| R^2^ | 0.84 | | 0.84 | | 0.84 | | 0.86 | |
| Term+SGA+LBW | | | | | | | | |
| Month 6 | -1.47 | 2.01 | -0.97 | 1.23 | -1.15 | 1.77 | -0.67 | 1.15 |
| Month 12 | -1.61 | 2.07 | -0.60 | 1.25 | -1.25 | 1.92 | -0.36 | 1.16 |
| Month 24 | -1.06 | 1.69 | -0.59 | 1.22 | -0.86 | 1.63 | -0.45 | 1.19 |
| Month 59 | 0.01 | 1.76 | -0.30 | 1.43 | -0.09 | 1.69 | -0.47 | 1.35 |
| R^2^ | 0.85 | | 0.85 | | 0.84 | | 0.84 | |
| Pretrm+AGA+LBW | | | | | | | | |
| Month 6 | -0.67 | 1.97 | -0.30 | 1.26 | -0.40 | 1.73 | -0.13 | 1.19 |
| Month 12 | -0.70 | 2.07 | 0.00 | 1.22 | -0.41 | 1.94 | 0.18 | 1.14 |
| Month 24 | -0.76 | 1.67 | -0.24 | 1.23 | -0.62 | 1.60 | -0.12 | 1.18 |
| Month 59 | 0.12 | 1.69 | -0.09 | 1.43 | 0.06 | 1.63 | -0.22 | 1.31 |
| R^2^ | 0.85 | | 0.86 | | 0.84 | | 0.85 | |
| Pretrm+SGA+LBW | | | | | | | | |
| Month 6 | -1.66 | 1.96 | -1.15 | 1.25 | -0.99 | 2.12 | -0.88 | 1.32 |
| Month 12 | -1.30 | 2.06 | -0.60 | 1.31 | -0.99 | 1.94 | -0.38 | 1.28 |
| Month 24 | -1.16 | 1.65 | -0.70 | 1.29 | -0.94 | 1.63 | -0.52 | 1.24 |
| Month 59 | -0.08 | 1.72 | -0.38 | 1.54 | -0.06 | 1.74 | -0.49 | 1.45 |
| R^2^ | 0.85 | | 0.86 | | 0.85 | | 0.86 | |
| ^1^SD = standard deviation; R^2^ = R-squared | | | | | | | | |


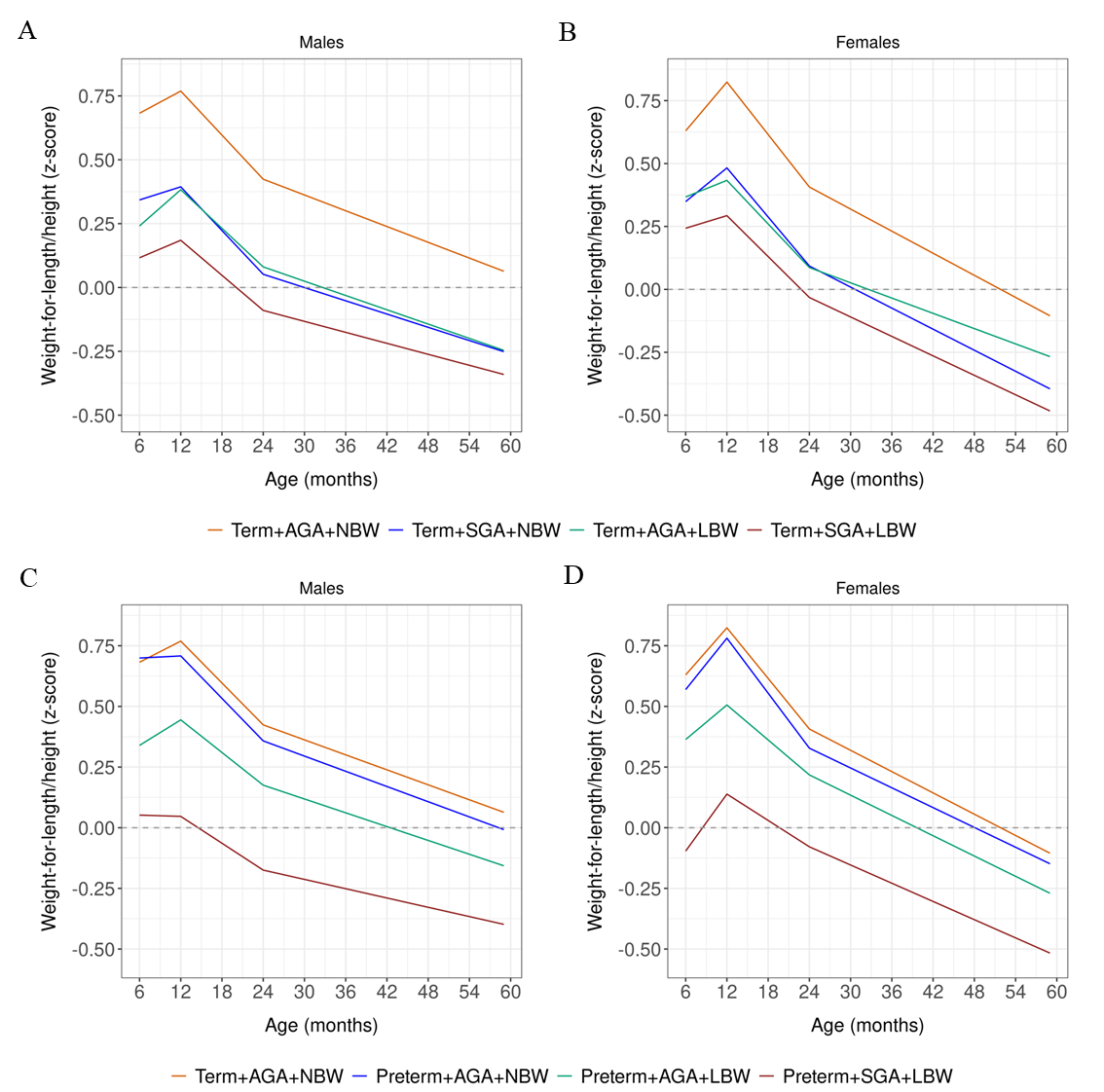


**Supplemental Figure 3:** Weight-for-Length/height growth trajectories (z-score) by vulnerability phenotype and sex from 6 to 59 months postnatal age. A) Growth trajectory for males (term); B) Growth trajectory for females (term); C) Growth trajectory for males (Term and Preterm); D) Growth trajectory for females (Term and Preterm)**;** Standard deviation (SD) 0,0 = median World Health Organization

| Supplemental Table 3: Weight-for-Length/height (z-scores) and change of z-scores (growth velocities) by vulnerability phenotype and sex, 2011-2017 (n= 1,993,068^1^) | | | | | | |
| --- | --- | --- | --- | --- | --- | --- |
| Vulnerability  phenotype | **Males** | | | **Females** | | |
|  | Weight-for-Length/height | | | Weight-for-Length/height | | |
|  | mean | SD^2^ | Δ^3^ | mean | SD^2^ | Δ^3^ |
| Term+AGA+NBW |  |  |  |  |  |  |
| Month 6 | 0.68 | 1.37 |  | 0.63 | 1.27 |  |
| Month 12 | 0.77 | 1.29 | 0.09 | 0.82 | 1.24 | 0.19 |
| Month 24 | 0.42 | 1.28 | -0.35 | 0.41 | 1.23 | -0.42 |
| Month 59 | 0.0 | 1.44 | -0.35 | -0.11 | 1.45 | -0.51 |
| R^2^ | 0.76 | |  | 0.77 | |  |
| Term+SGA+NBW |  |  |  |  |  |  |
| Month 6 | 0.34 | 1.47 |  | 0.35 | 1.32 |  |
| Month 12 | 0.39 | 1.30 | 0.05 | 0.48 | 1.26 | 0.13 |
| Month 24 | 0.05 | 1.27 | -0.34 | 0.09 | 1.24 | 0.39 |
| Month 59 | -0.25 | 1.43 | -0.30 | -0.40 | 1.43 | -0.49 |
| R^2^ | 0.75 | |  | 0.75 | |  |
| Preterm+AGA+NBW |  |  |  |  |  |  |
| Month 6 | 0.70 | 1.47 |  | 0.57 | 1.27 |  |
| Month 12 | 0.71 | 1.30 | 0.01 | 0.78 | 1.23 | 0.21 |
| Month 24 | 0.36 | 1.29 | -0.35 | 0.33 | 1.24 | -0.45 |
| Month 59 | -0.01 | 1.47 | -0.37 | -0.15 | 1.43 | -0.48 |
| R^2^ | 0.76 | |  | 0.76 | |  |
| Term+AGA+LBW |  |  |  |  |  |  |
| Month 6 | 0.24 | 1.26 |  | 0.37 | 1.19 |  |
| Month 12 | 0.38 | 1.25 | 0.14 | 0.43 | 1.24 | 0.07 |
| Month 24 | 0.08 | 1.24 | -0.30 | 0.09 | 1.24 | -0.35 |
| Month 59 | -0.25 | 1.44 | -0.33 | -0.27 | 1.45 | -0.35 |
| R^2^ | 0.75 | |  | 0.77 | |  |
| Term+SGA+LBW |  |  |  |  |  |  |
| Month 6 | 0.12 | 1.51 |  | 0.24 | 1.34 |  |
| Month 12 | 0.18 | 1.37 | 0.07 | 0.29 | 1.30 | 0.05 |
| Month 24 | -0.09 | 1.33 | -0.27 | -0.03 | 1.26 | -0.33 |
| Month 59 | -0.34 | 1.47 | -0.25 | -0.48 | 1.44 | -0.45 |
| R^2^ | 0.76 | |  | 0.76 | |  |
| Pretrm+AGA+LBW |  |  |  |  |  |  |
| Month 6 | 0.34 | 1.45 |  | 0.36 | 1.34 |  |
| Month 12 | 0.44 | 1.36 | 0.11 | 0.51 | 1.33 | 0.14 |
| Month 24 | 0.18 | 1.33 | -0.27 | 0.22 | 1.27 | -0.29 |
| Month 59 | -0.16 | 1.55 | -0.33 | -0.27 | 1.49 | -0.49 |
| R^2^ | 0.78 | |  | 0.78 | |  |
| Pretrm+SGA+LBW |  |  |  |  |  |  |
| Month 6 | 0.05 | 1.42 |  | -0.10 | 1.54 |  |
| Month 12 | 0.05 | 1.40 | -0.01 | 0.14 | 1.38 | 0.23 |
| Month 24 | -0.17 | 1.35 | -0.22 | -0.08 | 1.29 | -0.23 |
| Month 59 | -0.40 | 1.62 | -0.22 | -0.52 | 1.59 | -0.44 |
| R^2^ | 0.78 | |  | 0.79 | |  |
| ^1^ 1,993,068 children aged 6-59 months, with two or more weight and height measurements evaluated between 2011 and 2017 were included in the study. We excluded 2,620 children with biologically implausible z-scores according to the WHO cutoffs for weight-for-length /height z-scores (WLZ/WHZ <-5 and >5), and 26,310 children with only one measurement of W /LH during the study period.  ^1^ SD = standard deviation.  ^2^change (Δ) in the mean z-score between two-time points. | | | | | | |


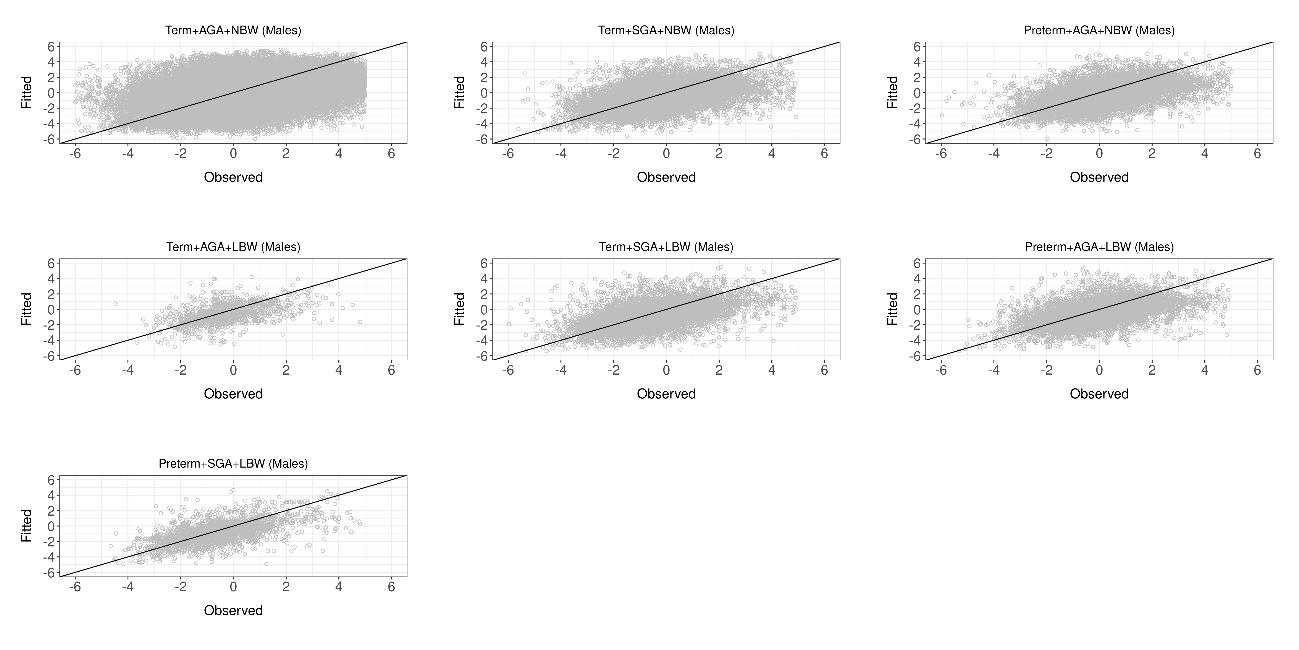


**Supplemental Figure 4**: Observed versus predicted values for length/height-for-age (z-score) for males.


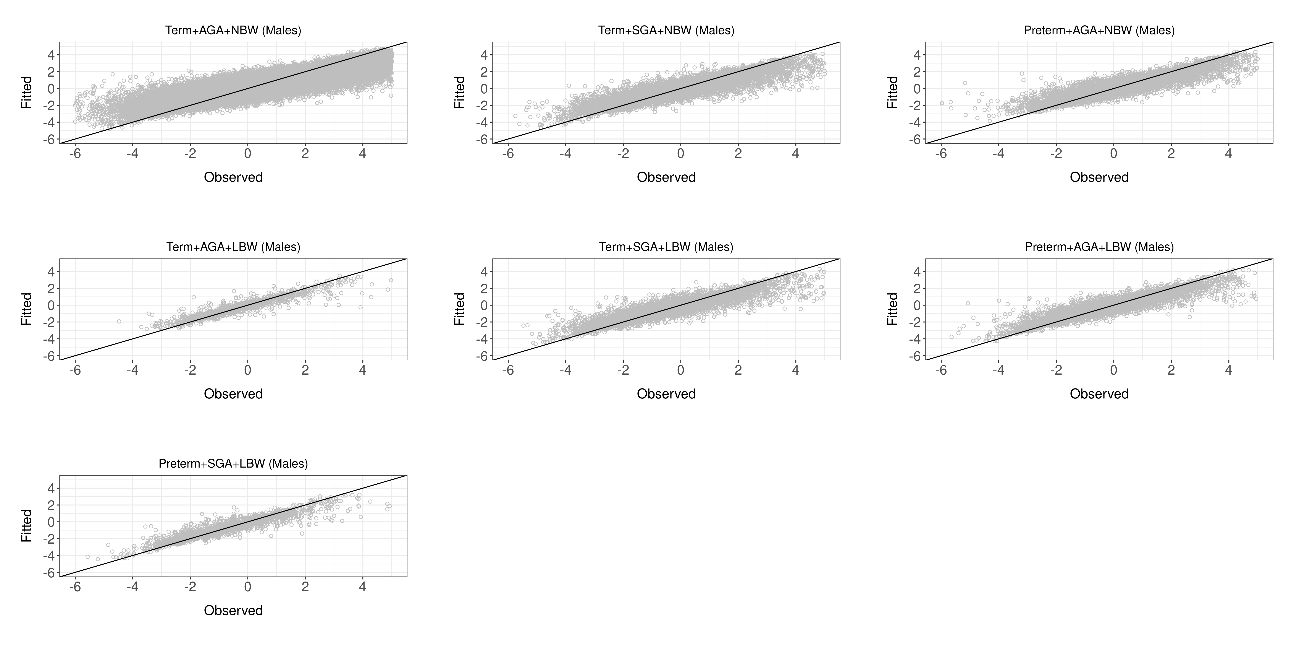


**Supplemental Figure 5:** Observed versus predicted values for weight-for-age (z-score) for males.


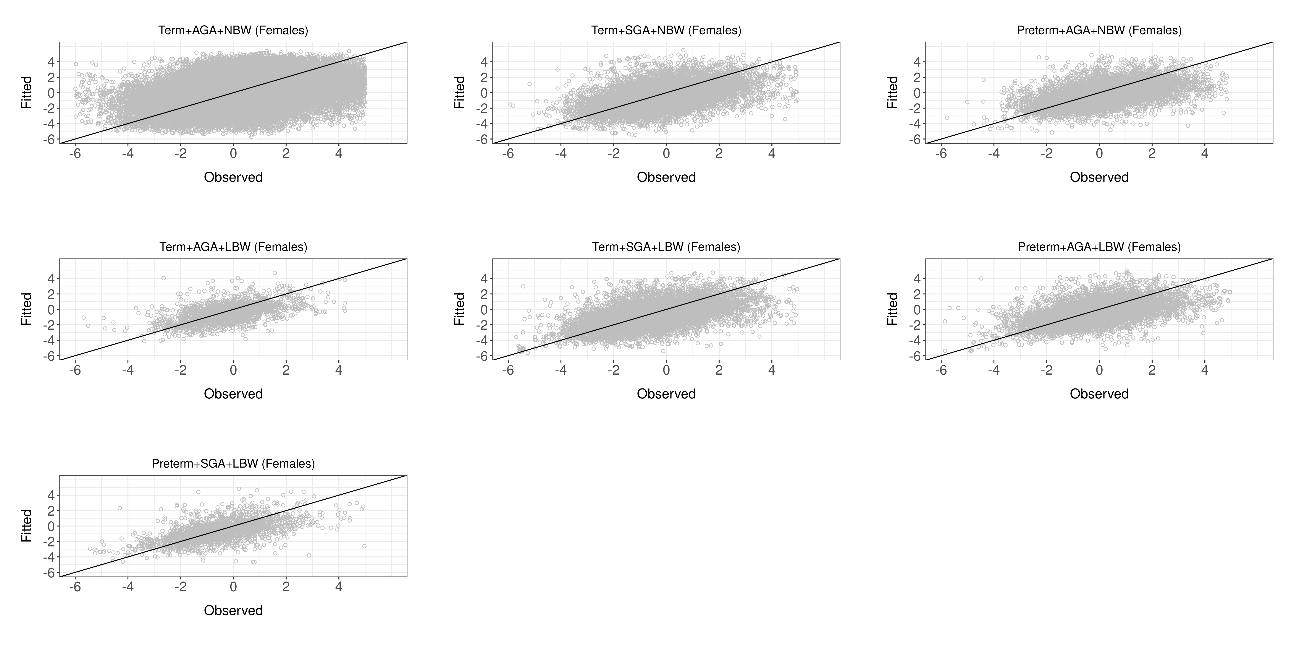


**Supplemental Figure 6**: Observed versus predicted values for length/height-for-age (z-score) for females.


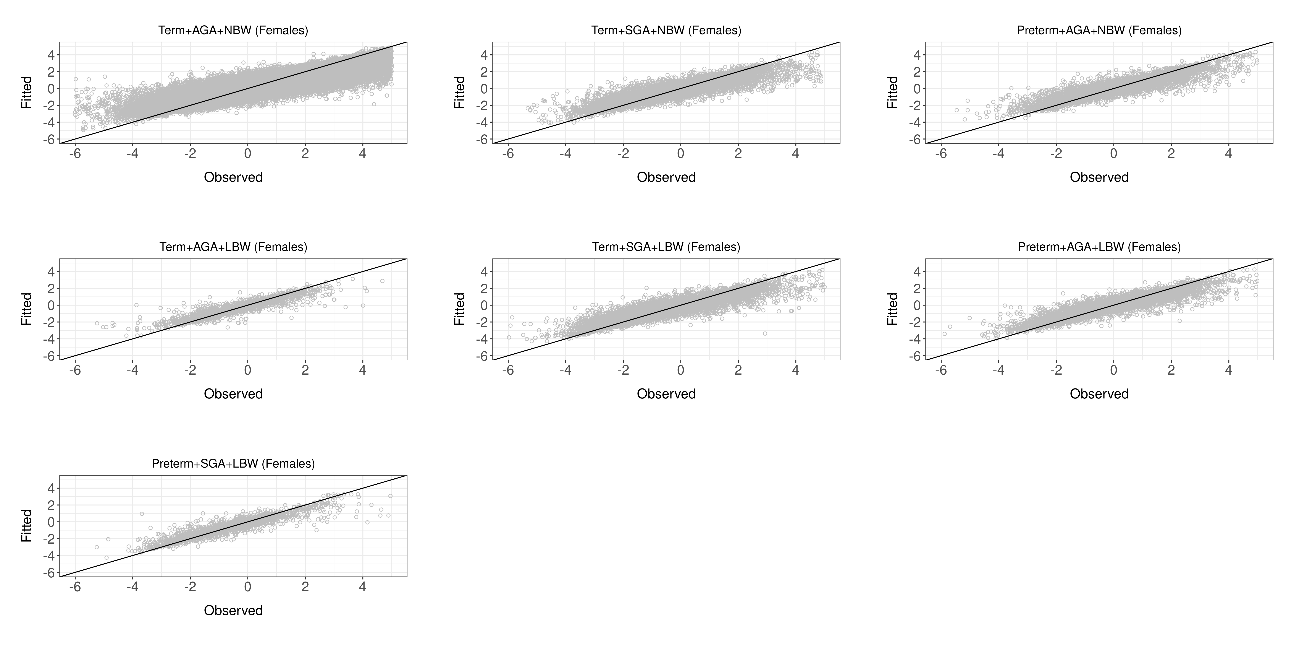


**Supplemental Figure 7**: Observed versus predicted values for weight-for-age (z-score) for females*.*

*Supplementary analysis*

**Supplemental Figure 8.** Study population flow diagram, 2011-2017.

^1^Live births large for gestational age (LGA) > 90th percentile by INTERGROWTH-21st); ^2^records with biologically implausible z-scores according to the WHO cutoffs for weight-for-age (WAZ) (z-scores < -6 and > 5), length/height for age ( L/HAZ) ( z scores < -6 and > 6); ^3^preterm birth - gestational age < 37 completed weeks, ^4^term -gestational age between 37-42 weeks; ^5^low birth weight (LBW) - birth weight < 2500 g; ^6^normal birth weight (NBW) - birth weight between 2500-6500 g; ^7^small for gestational age (SGA) - birth weight for gestational age <10^th^ percentile of the INTERGROWTH-21^st^; ^8^adequate for gestational age (AGA) - birth weight for gestational age between the 10th and 90th percentiles.


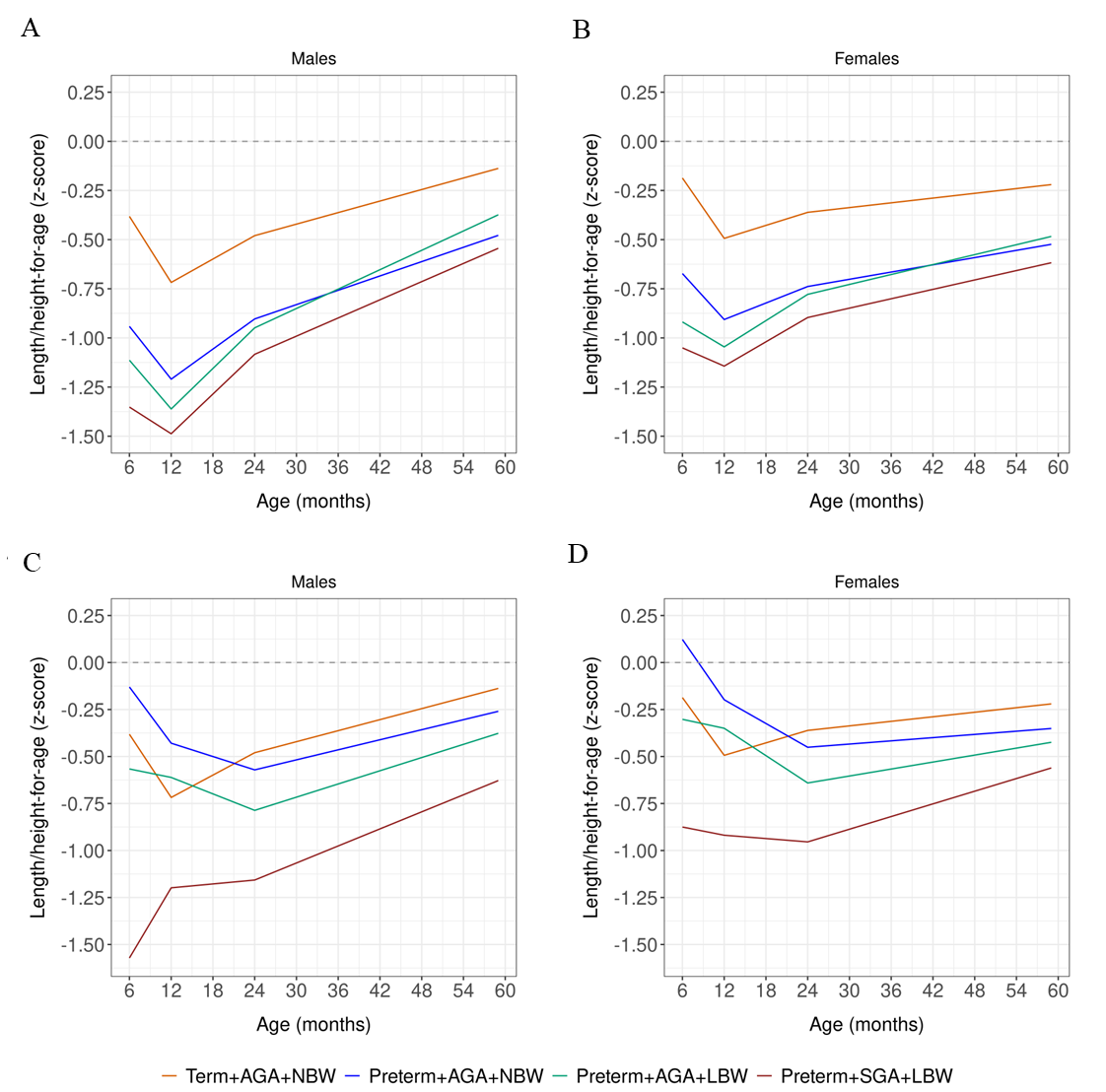


**Supplemental Figure 9:** Length/height-for-age growth trajectories (z-score) by vulnerability phenotype and sex from 6 to 59 months postnatal age. A) Growth trajectory for males (Term); B) Growth trajectory for females (Term); C) Growth trajectory for males (Term and Preterm); D) Growth trajectory for females (Preterm)**;** Standard deviation (SD) 0,0 = median World Health Organization


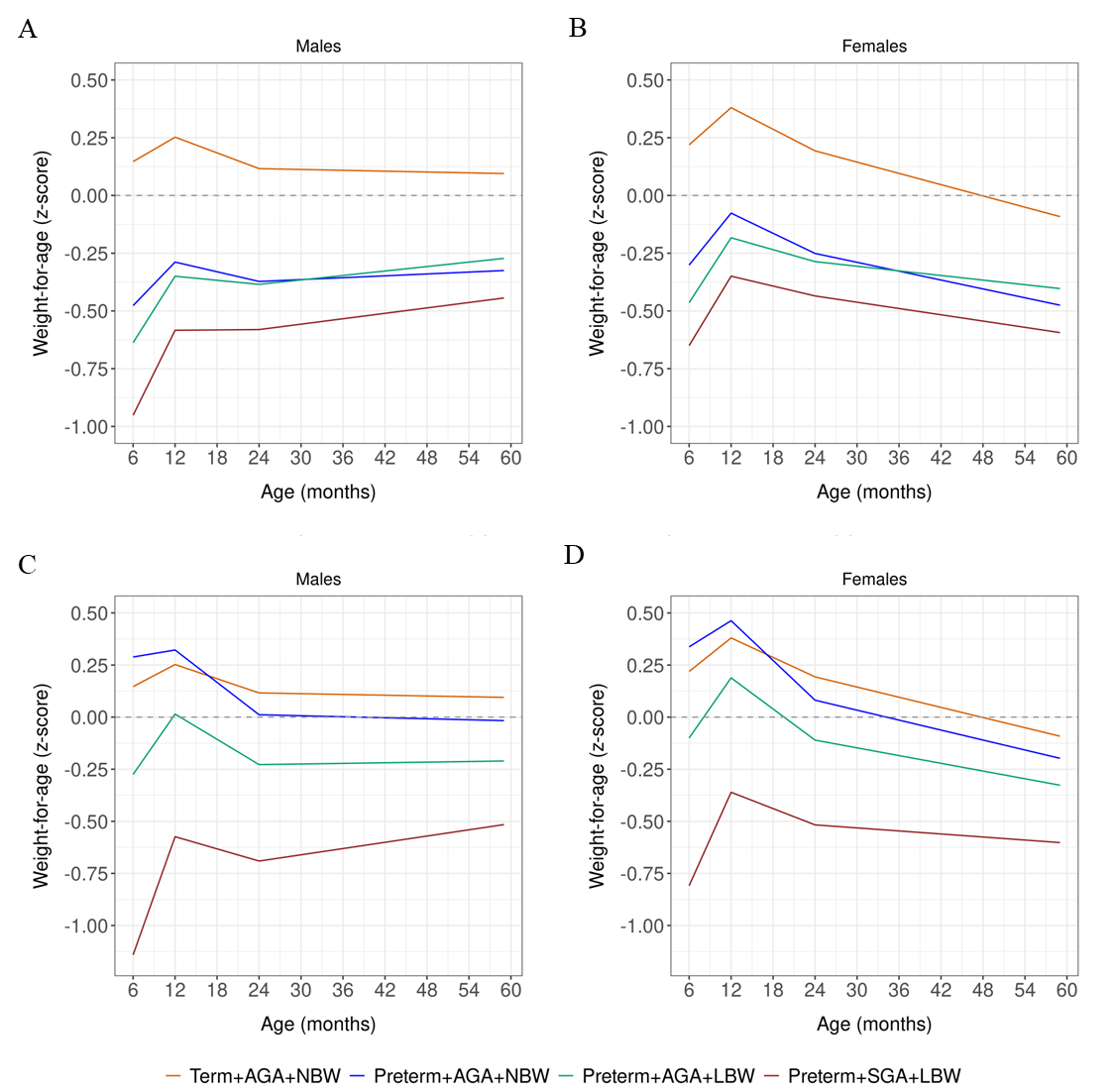


**Supplemental Figure 10:** Weight-for-age growth trajectories (z-score) by vulnerability phenotype and sex from 6 to 59 months postnatal age. A) Growth trajectory for males (Term); B) Growth trajectory for females (Term); C) Growth trajectory for males (Term and Preterm); D) Growth trajectory for females (Term and Preterm)**;** Standard deviation (SD) 0,0 = median World Health Organization

| **Supplemental Table 4**: Longitudinal anthropometric indicators (z-scores) by vulnerability phenotype and sex, 2011-2017 (2,095,236) | | | | | | | | |
| --- | --- | --- | --- | --- | --- | --- | --- | --- |
| **Vulnerability**  **phenotype** | **Males** | | | | **Females** | | | |
|  | Length/height-for-age | | Weight-for-age | | Length/height-for-age | | Weight-for-age | |
|  | mean | SD | mean | SD | mean | SD | mean | SD |
| Term+AGA+NBW | | | | | | | | |
| Month 6 | -0.38 | 1.55 | 0.15 | 1.07 | -0.19 | 1.41 | 0.22 | 0.99 |
| Month 12 | -0.72 | 1.63 | 0.25 | 1.08 | -0.49 | 1.52 | 0.38 | 0.98 |
| Month 24 | -0.48 | 1.32 | 0.12 | 1.09 | -0.36 | 1.29 | 0.19 | 1.06 |
| Month 59 | -0.14 | 1.13 | 0.09 | 1.22 | -0.22 | 1.13 | -0.09 | 1.16 |
| R^2^ | 0.65 | | 0.78 | | 0.65 | | 0.79 | |
| Term+SGA+NBW | | | | | | | | |
| Month 6 | -0.94 | 1.51 | -0.48 | 1.04 | -0.67 | 1.40 | -0.30 | 0.98 |
| Month 12 | -1.21 | 1.66 | -0.29 | 1.07 | -0.91 | 1.59 | -0.08 | 1.02 |
| Month 24 | -0.90 | 1.33 | -0.37 | 1.09 | -0.74 | 1.30 | -0.25 | 1.08 |
| Month 59 | -0.48 | 1.16 | -0.32 | 1.20 | -0.52 | 1.14 | -0.48 | 1.17 |
| R^2^ | 0.65 | | 0.77 | | 0.65 | | 0.78 | |
| Preterm+AGA+NBW | | | | | | | | |
| Month 6 | -0.13 | 1.66 | 0.29 | 1.12 | 0.12 | 1.41 | 0.34 | 0.97 |
| Month 12 | -0.43 | 1.69 | 0.32 | 1.10 | -0.20 | 1.62 | 0.46 | 1.00 |
| Month 24 | -0.57 | 1.35 | 0.01 | 1.12 | -0.45 | 1.30 | 0.08 | 1.09 |
| Month 59 | -0.26 | 1.18 | -0.02 | 1.26 | -0.35 | 1.14 | -0.20 | 1.19 |
| R^2^ | 0.67 | | 0.79 | | 0.65 | | 0.79 | |
| Term+AGA+LBW | | | | | | | | |
| Month 6 | -1.11 | 1.37 | -0.64 | 1.10 | -0.92 | 1.33 | -0.46 | 0.95 |
| Month 12 | -1.36 | 1.72 | -0.35 | 1.07 | -1.05 | 1.59 | -0.18 | 1.03 |
| Month 24 | -0.95 | 1.24 | -0.38 | 1.08 | -0.78 | 1.32 | -0.29 | 1.07 |
| Month 59 | -0.37 | 1.19 | -0.27 | 1.27 | -0.48 | 1.18 | -0.40 | 1.20 |
| R^2^ | 0.66 | | 0.78 | | 0.67 | | 0.79 | |
| Term+SGA+LBW | | | | | | | | |
| Month 6 | -1.35 | 1.73 | -0.95 | 1.17 | -1.05 | 1.46 | -0.65 | 1.09 |
| Month 12 | -1.49 | 1.73 | -0.58 | 1.18 | -1.14 | 1.60 | -0.35 | 1.11 |
| Month 24 | -1.08 | 1.40 | -0.58 | 1.16 | -0.90 | 1.35 | -0.44 | 1.14 |
| Month 59 | -0.54 | 1.21 | -0.44 | 1.29 | -0.62 | 1.19 | -0.59 | 1.23 |
| R^2^ | 0.68 | | 0.79 | | 0.66 | | 0.79 | |
| Pretrm+AGA+LBW | | | | | | | | |
| Month 6 | -0.57 | 1.65 | -0.27 | 1.20 | -0.30 | 1.38 | -0.10 | 1.13 |
| Month 12 | -0.61 | 1.73 | 0.01 | 1.17 | -0.35 | 1.61 | 0.19 | 1.10 |
| Month 24 | -0.79 | 1.39 | -0.23 | 1.18 | -0.64 | 1.34 | -0.11 | 1.13 |
| Month 59 | -0.38 | 1.22 | -0.21 | 1.32 | -0.42 | 1.19 | -0.33 | 1.22 |
| R^2^ | 0.68 | | 0.81 | | 0.66 | | 0.80 | |
| Pretrm+SGA+LBW | | | | | | | | |
| Month 6 | -1.57 | 1.68 | -1.14 | 1.20 | -0.88 | 1.83 | -0.81 | 1.32 |
| Month 12 | -1.20 | 1.82 | -0.57. | 1.26 | -0.92 | 1.58 | -0.36 | 1.21 |
| Month 24 | -1.16 | 1.42 | -0.69. | 1.24 | -0.95 | 1.37 | -0.52 | 1.19 |
| Month 59 | -0.63 | 1.25 | -0.52 | 1.42 | -0.56 | 1.17 | -0.60 | 1.37 |
| R^2^ | 0.70 | | 0.82 | | 0.68 | | 0.82 | |
| SD= standard deviation; R^2^=R-squared | | | | | | | | |

| **Supplemental Table 5:** Change of z-scores (growth velocities) for length/height-for-age and weight-for-age by vulnerability phenotype and sex, 2011-2017 (2,095,236) | | | | |
| --- | --- | --- | --- | --- |
| **Vulnerability**  **phenotype** | **Males** | | **Females**^1^ | |
|  | Length/height-for-age | Weight-for-age | Length/height-for-age | Weight-for-age |
|  | Δ^1^ | Δ^1^ | Δ^1^ | Δ |
| Term+AGA+NBW | |  |  |  |
| 6-12 months | -0.34 | 0.11 | -0.31 | 0.16 |
| 12-24 months | 0.24 | -0.14 | 0.13 | -0.19 |
| 24-58 months | 0.34 | -0.02 | 0.14 | -0.28 |
| Term+SGA+NBW | |  |  |  |
| 6-12 months | -0.27 | 0.19 | -0.2 | 0.22 |
| 12-24 months | 0.31 | -0.08 | 0.17 | -0.17 |
| 24-58 months | 0.43 | 0.05 | 0.21 | -0.22 |
| Preterm+AGA+NBW | |  |  |  |
| 6-12 months | -0.30 | 0.03 | -0.32 | 0.13 |
| 12-24 months | -0.14 | -0.31 | -0.25 | -0.38 |
| 24-58 months | 0.31 | -0.03 | 0.10 | -0.28 |
| Term+AGA+LBW | |  |  |  |
| 6-12 months | -0.25 | 0.29 | -0.13 | 0.28 |
| 12-24 months | 0.41 | -0.04 | 0.27 | -0.10 |
| 24-58 months | 0.58 | 0.11 | 0.29 | -0.12 |
| Term+SGA+LBW | |  |  |  |
| 6-12 months | -0.14 | 0.37 | -0.09 | 0.30 |
| 12-24 months | 0.40 | 0.00 | 0.25 | -0.09 |
| 24-58 months | 0.54 | 0.14 | 0.28 | -0.16 |
| Preterm+AGA+LBW | |  |  |  |
| 6-12 months | -0.04 | 0.29 | -0.05 | 0.29 |
| 12-24 months | -0.18 | -0.24 | -0.29 | -0.30 |
| 24-58 months | 0.41 | 0.02 | 0.22 | -0.22 |
| Preterm+SGA+LBW | |  |  |  |
| 6-12 months | 0.37 | 0.57 | -0.04 | 0.45 |
| 12-24 months | 0.04 | -0.12 | -0.04 | -0.16 |
| 24-58 months | 0.53 | 0.17 | 0.39 | -0.08 |
| ^1^ change **(**Δ) in the mean z-score between two-time points. | | | | |
